# Double Uterus and Vagina

**Published:** 1897-02

**Authors:** Frank A. Jones

**Affiliations:** 155 Lake Avenue. Rochester, N. Y.


### Correspondence.

### DOUBLE UTERUS AND VAGINA.

ROCHESTER, N. Y., January 14, 1897.

*To the Editor :*

SIR—I wish to report an anomalous case, viz.: Miss L., 25 years of age, came to me complaining of painful menstruation, which came regularly every twenty-eight days and continued for five or six days. She had been treated by several physicians. On examination I found two well-formed uteri with cervices perfectly normal, excepting a slight stenosis of the left cervical canal, two vaginæ, the left lying closely to the left wall, or in other words, collapsed, although perfect. The left uteri inclined to the left side so much so that I at first diagnosticated it as an enlarged ovary. She was wholly unconscious of her condition.

FRANK A. JONES.

155 LAKE AVENUE.

